# SARS-CoV-2. Long Distance Airborne Transmission and its Public Health Implications

**DOI:** 10.6061/clinics/2020/e2343

**Published:** 2020-11-02

**Authors:** Gustavo Silveira Graudenz, Cristiane Degobbi, Paulo Hilario Saldiva

**Affiliations:** I Universidade Santo Amaro (UNISA), Saude do Idoso III. Modulo de ensino - Disciplina: Reumatologia e Imunologia, Sao Paulo, SP, BR; II Departamento de Microbiologia. Universidade Santo Amaro (UNISA), Sao Paulo, SP, BR; III Departamento de Patologia. Universidade de Sao Paulo. Hospital das Clinicas (HCFMUSP), Sao Paulo, SP, BR

By the end of July 2020, more than 15 million confirmed cases of and more than half a million deaths from coronavirus disease (COVID-19), caused by severe acute respiratory syndrome coronavirus 2 (SARS-CoV-2), had been recorded. Of these, more than 2.5 million cases and 85,000 deaths were recorded in Brazil. As it is difficult to sustain measures of social isolation, more focused and effective preventive measures against viral transmission are urgently needed to prevent the spread of the disease. Until recently, the World Health Organization (WHO) endorsed only two modes of viral transmission. The first mode is via inhalation of large respiratory droplets generated from coughing, sneezing, or breathing by an infected individual, wherein the infective potential is short-ranged. The second mode is via direct contact with contaminated surfaces (fomites), wherein the virus is carried by the hands to the nose, mouth, or eyes.

Considering this, frequent hand washing, maintaining a social distance of 1 m, good respiratory hygiene, and avoiding crowds (no specifications) are considered main preventive measures (1).

Until now, COVID-19 has not been considered an airborne disease. The only diseases recognized as belonging to this category are tuberculosis *(Mycobacterium tuberculosis)*, chicken pox (*varicella-zoster virus*), measles (*Morbillivirus*), and invasive aspergillosis (*Aspergillus* sp.) (2). The category is restricted to diseases transmitted by small viable and infectious particles that are able to travel long distances, stay for long periods in suspension, are strongly influenced by air currents, and are able to penetrate deep into the lungs of individuals who have had no previous direct contact with or have not been in close proximity to an infected individual. Despite some controversies regarding the penetration of particles into the respiratory system, it is widely accepted that small particles with an aerodynamic diameter of less than 10 μm are able to pass the glottic barrier and penetrate up to the thoracic level. Particles ranging from 5 to 10 μm have the potential to transmit disease over both long and short distances, and particles less than 5 μm in diameter can penetrate up to the alveolar level (3). In contrast, particles larger than 20 μm in diameter have a more ballistic behavior, meaning they are more under the influence of gravity than wind currents.

The particles generated through breathing, coughing, and sneezing are between 4 and 8 µm in diameter (4). More recent and precise studies that take into account the age-dependent decay process of the particles report that most exhaled particles measure less than 1 µm and are generated during speaking and not only during coughing or sneezing (5). In addition, exhaled particles are not necessarily isolated in muco-salivary droplets. During the exhalation process, multiphase turbulent gas clouds are created.

These clouds retain their temperature and humidity and allow the contained droplets to evade evaporation for much longer than isolated droplets can. Under these conditions, the lifetime of a droplet could be considerably extended, by a factor of up to 1000, from a fraction of a second to minutes. As a result, these clouds can carry particles 7 or 8 m away from the infected individual, after which the particles undergo dehydration and aging, resulting in smaller particles that are able to travel even longer distances (6).

According to Roy and Milton (7), airborne infections can be largely underestimated because of methodological limitations. They suggest the following classification for airborne diseases:

1. Mandatory: when the pathogen can only be transmitted through the airborne route (*e.g.*, *tuberculosis*).
2. Preferential: when the pathogen can be transmitted by other routes but the airborne route is the main route (*e.g.*, measles, smallpox).
3. Opportunistic: when the pathogen is mainly transmitted by other routes but can be transmitted by the airborne route under certain circumstances.

Currently, there is no clear evidence of airborne transmission of SARS-CoV-2. This is a matter of intense debate. Its predecessor, SARS-CoviD-1, the agent that caused Severe Acute Respiratory Syndrome (SARS) in Hong Kong in 2003, showed strong evidence of opportunistic airborne transmission in different environments, such as collective housing environments (8), indoor environments such as airplanes (9), and health service institutions (10).

Similarly, some researchers considered airborne transmission of another coronavirus, Middle East Respiratory Syndrome coronavirus (MERS-CoV), which has been described as unable to infect the upper airways but as having a preferential affinity toward the lower airway, with infective particles less than 10 µm in diameter (11).

The consideration of airborne transmission of SARS-CoV-2 by the (WHO) was based on evidence from a study by Ong et al. (12) that suggested transmission of SARS-CoV-2 through infected surfaces and contaminated individual protection equipment as well as long distance environment contamination. The negative results from the air samples were interpreted as effective room disinfection and air renovation measures. Despite the air samples testing SARS-CoV-2-negative, samples from the recirculation system tested positive, revealing that small particles were carried over longer distances.

Considering this, new studies have been conducted, and evidence of airborne transmission has started increasing. In a hospital designated to treat patients with COVID-19, airborne viruses were detected in sub-micrometric particles and were more concentrated in used protective apparel of health professionals (13). In another study in a hospital treating COVID-19 patients in Nebraska, USA, the largest viral concentrations were detected in the ventilation grills, indirectly indicating long-distance infective particle transportation into the building ventilation system (14). In an experimental study conducted in an aerosol chamber, potentially infective and stable particles lasting for up to 3 h were identified in a controlled contamination experiment using particles with less than 5 microns in diameter (15). Considering the reported evidence and the many similarities among these coronaviruses (SARS-CoV-1, MERS-CoV), it is likely that SARS-CoV-2 is transmitted via the air. However, some questions remain unanswered, for instance, the origin and area as well as the infectious dose and infection rates after exposure to particles smaller than 10 µm (16).

Despite the controversies, experts recommend precaution in all indoor environment-related situations. Measures such as increasing air exchange rates, avoiding air recirculation, downwind positioning relative to other individuals, and reducing the number of people sharing the same indoor environment should be considered, among the general measures aimed at reducing the infection risk (17). In health care settings, the Center for Disease Control’s recommendations for prevention of airborne transmission include maintaining a negative pressure environment, fine filtering of exhaust air from infected patients’ rooms, maintaining high air exchange rates (12 air exchanges per hour), shutting recirculation ducts, and establishing pressure cascades (2) in these settings until further evidence of long distance transmission is obtained

Unfortunately, these precautionary measures have not yet been applied in most health care facilities in Brazil. Even when it is not feasible to establish negative pressure or pressure cascades using ventilation systems, a simple alternative could be to use natural ventilation to increase air exchange rates and thus ensure pathogen dilution. It is important to avoid the use of a ventilation system without air exchange such as split systems in these settings. In our scenario, scientific data reinforces that indoor air quality standards for health care in Brazil, the NBR 7256 (18), must be updated to protect health personnel dealing with diseases with a high potential for being contagious, such as COVID-19. Recently, a call for action supported by 239 scientists from different parts of the world was published that indicated that information on warnings and precautionary measures for the control of potential aerial transmission of SARS-CoV-2 should be conveyed by health authorities (19). Considering the risk of airborne transmission of COVID-19 and taking its preventive measures is particularly important at this moment when several countries, including Brazil, are progressively increasing their economic and social activities and gathering people is inevitable. So, it is important to stress the necessity of maximizing air renewing and mandatory use of face masks, not only in health care settings but also in commonly shared indoor environments, like workplaces, schools and public transportation.
